# First records of *Aedes pulcritarsis* (Rondani, 1872) (Diptera: Culicidae) in Austria

**DOI:** 10.1007/s00436-022-07430-w

**Published:** 2022-01-15

**Authors:** Karin Bakran-Lebl, Hans Jerrentrup, Eleni Daroglou, Wolf Peter Pfitzner, Hans-Peter Fuehrer, Franz Allerberger

**Affiliations:** 1 Institute for Medical Microbiology & Hygiene, AGES - Austrian Agency for Health and Food Safety Ltd, Währinger Straße 25A, 1090 Vienna, Austria; 2 Institute of Parasitology, Vetmeduni Vienna, Veterinaerplatz 1, 1210 Vienna, Austria; 3 Verein Biologische Gelsenregulierung March-Thaya Auen, Rathausplatz 1, 2273 Hohenau an der March, Austria; 4 KABS - Kommunale Aktionsgemeinschaft Zur Bekämpfung Der Schnakenplage E.V, 67346 Speyer, Germany

**Keywords:** *Aedes pulcritarsis*, Austria, Mosquitoes, Species inventory

## Abstract

*Aedes pulcritarsis* is a tree-hole breeding species with its main distribution in the Mediterranean area. Within the scope of two independent monitoring programmes, this mosquito species was detected for the first time in Austria, in the province of Lower Austria (2018, districts Mistelbach and Gaenserndorf; 2020, district Bruck an der Leitha). As the climatic and habitat situation in Central Europe seems to be generally suitable for this species, the most likely explanation for the species not being recorded previously is that it might have been overlooked in the past due to its specialized breeding habitat. However, further research on the distribution of *Ae. pulcritarsis* in Austria would be needed to support this hypothesis. The results from this study will contribute to the investigation of the northern distribution limit of *Ae. pulcritarsis* in Europe and possible changes thereof.

## Introduction

*Aedes* (*Ochlerotatus*) *pulcritarsis* (Rondani, 1872) (in some literature incorrectly spelled *Ae. pulchritarsis*) is a species of the Western Palaearctic, with its main distribution in the Mediterranean region. It has been documented in the following countries/regions: Albania, Algeria, Azerbaijan, Bulgaria, Croatia, Czech Republic, France (incl. Corsica), Georgia, Greece, Hungary, Israel, Italy (incl. Sicily), Kosovo, Lebanon, Moldova, Montenegro, Morocco, Portugal, Romania, Russia (south-western incl. Crimean Peninsula), Serbia, Slovakia, Spain (incl. Balearic Islands), Tunisia and Turkey (Robert et al. 2019) and Iran (Azari-Hamidian 2007). Additionally, in 2016, a single specimen was reported from a rural area near a fragmented forest in the federal state of Rhineland-Palatinate in Germany (Kampen et al. 2017).

*Aedes pulcritarsis* uses phytotelms, especially tree-holes, as its preferred breeding sites. A study conducted in Israel found *Ae. pulcritarsis* breeding in tree-holes of oaks (*Quercus calliprinos*, *Q. boissieri*, *Q. ithaburensis*), planes (*Platanus palestinensis*, *P. orientalis*) and eucalyptus (*Eucalyptus* sp.). Breeding sites were only found in shady areas of woodlands with mature trees, located in forested mountainous areas with high annual precipitation (Müller et al. 2012). The temperature of the water in the breeding sites never exceeds 21 °C and larval development may take 2 months (Becker et al. 2020). *Aedes pulcritarsis* usually has two generations per year (Becker et al. 2020). In contrast to many other mosquito species preferring small water hollows as breeding sites, such as *Ae. albopictus*, *Ae. japonicus* or *An. plumbeus*, there are no records of this species breeding in tyres, road drains or rock pools. Although they generally seem to avoid man-made habitats, larvae have been observed in wooden buckets (European Centre for Disease Prevention and Control 2012; Müller et al. 2012).

Here, we report the first findings of *Ae. pulcritarsis* in Austria.

## Material and methods

**Fig. 1:**
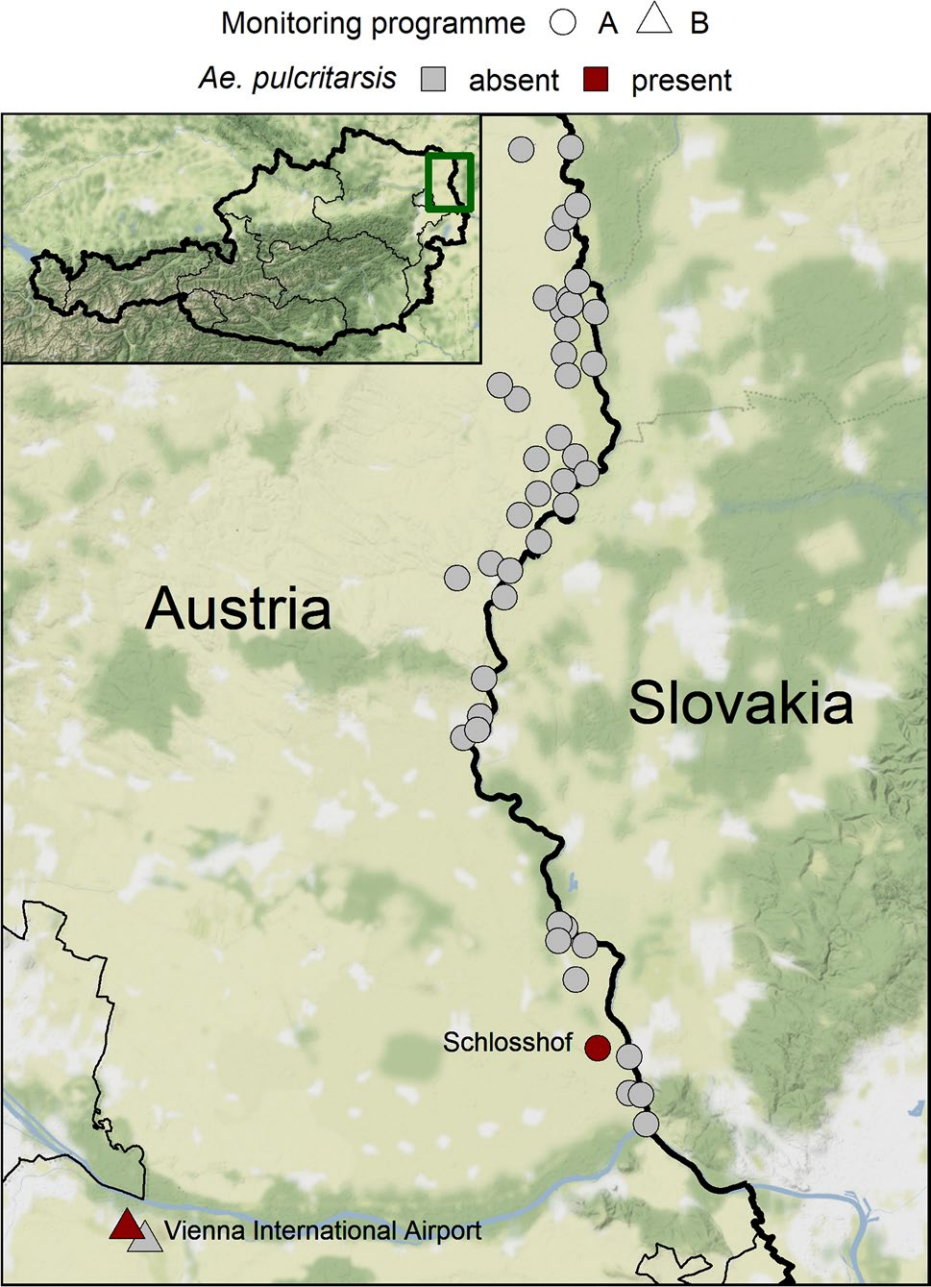
Locations sampled in the framework of the mosquito monitoring programmes. While the sites were sampled irregularly (depending on flood situation) in monitoring A, regular checks were carried out at weekly intervals in monitoring B. Map tiles by Stamen Design, under CC BY 3.0. Data by OpenStreetMap, under ODbL. Data source borders: NUTS units, Statistik Austria—data.statistik.gv.at

Individuals of *Ae. pulcritarsis* were captured within the scope of two independently conducted mosquito monitoring programmes (Fig. 1; created using program R (R Core Team 2021)). Both programmes were conducted within the same province, Lower Austria, but represent quite different habitats.

### Mosquito monitoring A

Mosquito monitoring A is conducted in the most eastern part of Austria in the floodplains of the transboundary rivers March/Morava and Thaya/Dyje (March-Thaya-Ramsar Wetlands; within the districts Mistelbach and Gaenserndorf). The aim of this programme is to monitor the effectiveness of mosquito control measures implemented in this area. Since 2011, EVS mosquito traps (BioQuip Products, Inc, Rancho Dominguez, USA), using CO_2_ from dry ice as attractant, have been in operation at 35–40 locations on the floodplains of these rivers and the neighbouring villages. The investigated area ranges from Rabensburg (48.6819 N, 16.9193E) to the river March/Morava at its junction with the Donau/Danube (48.1742 N, 16.9757 E). Traps are active for one night once or twice a month (depending on flooding events) from April to September. In Schlosshof, where the *Ae. pulcritarsis* specimens were found, the sampling site is surrounded by an extended park area with dense forest consisting of very old trees.

### Mosquito monitoring B

Mosquito monitoring B is conducted at the Vienna International Airport (48.111°N, 16.569°E, 183 m a.s.l.) to detect the possible introduction of alien mosquito species via air travel. The airport is located near the city of Vienna, in the district of Bruck an der Leitha. The airport is located at the north-western edge of the Pannonian biogeographic region, which is characterized by a humid continental climate. Since 2018, sampling has been taking place in a green courtyard, approximately 70 m from the airport’s movement area. In this courtyard, a BG-Sentinel 2 (Biogents AG, Regensburg, Germany), equipped with a CO_2_ release and a specific lure (BG-Sweetscent), is set up from the beginning of May to the end of October. The trap is operated continuously, and captured mosquitoes are collected weekly (Bakran-Lebl et al., 2021a).

### Mosquito identification

Female mosquitoes were identified to species level by morphological characteristics using the keys of Gunay et al. (2018) and Becker et al. (2020).

The morphological characteristics of *Ae. pulcritarsis* are very similar to *Ae. berlandi*, as females differ only slightly in the scutal colouration pattern (Becker et al. 2020). Thus, additional identification by DNA barcoding was conducted. For this, DNA was extracted from one leg of each individual. To this end, three 1.4-mm ceramic beads (Precellys Ceramic Kit 2.8 mm, Peqlab, Erlangen, Germany) were added to each tissue sample. Homogenization was performed with a TissueLyser II (Qiagen, Hilden, Germany). Afterwards, DNA was extracted using a blood and tissue DNA isolation kit (DNeasy®, Qiagen, Hilden, Germany) according to the manufacturer’s instructions. Conventional polymerase chain reactions (PCRs), targeting the barcode region within the mitochondrial cytochrome c oxidase subunit I gene (COI), using primers H15CuliCOIFw and H15CuliCOIRv as well as LCO1490 and HCO2198, were performed as reported previously (Folmer et al. 1994; Werblow et al. 2016). PCR products were sequenced at LGC Genomics GmbH, Germany. Resulting sequences were compared to sequences available in GenBank® and BOLD systems databases. Sequences were uploaded to GenBank® (MZ457071, MZ457072, OK076908). For one specimen captured within mosquito monitoring A, the genetic identification was confirmed in a second laboratory, at KABS e.V. (Speyer, Germany).

## Results

A female *Ae. pulcritarsis* (MZ457071) was found in the course of mosquito monitoring A during the night of 25 to 26 July 2018 at the parking area of the “Schlosshof” estate (48.2163 N, 16.9351 E). After this first catch, a more intensive trapping was conducted at this site, resulting in the capture of a further female during the night of 9 to 10 August 2018 (OK076908). Approximately 30 km further away, within the area of mosquito monitoring B, a single female *Ae. pulcritarsis* (MZ457072) was found in the catch from 5 to 12 August 2020 (Fig. 1).

The sequences of the two individuals from mosquito monitoring A were identical but they differed by two nucleotides from the specimen captured during mosquito monitoring B. However, all three sequences are in the same mitochondrial COI gene cluster as specimens collected in Turkey and Greece.

## Discussion

The discovery of a new species in a country always raises the question of why it has not been recorded before. Basically, there are three explanations: first, it was always present, but has not yet been found, either due to limited sampling effort during mosquito monitoring, low attractiveness of commonly used trapping methods and/or species rarity. This was likely the case for *Orthopodomyia pulcripalpis,* recorded for the first time in Austria in 2016 (Zittra et al. 2017). Second, the species could have been recently introduced by active spread from neighbouring countries because of changing climatic and environmental conditions. Examples of Mediterranean species actively invading Austria are *Anopheles hyrcanus* (first record 2012, Lebl et al. 2013), a species, which has since repeatedly been found in this country (Lebl et al. 2015), and *Culiseta longiareolata* (Seidel et al. 2013). Third, the species could have been recently introduced by anthropogenic activities such as freight transports. This was the main introduction route into Europe for container-breeding species such as *Ae. albopictus*, *Ae. japonicus* and *Ae. koreicus*, which have also been reported for Austria (Seidel et al. 2012; Fuehrer et al. 2020; Bakran-Lebl et al., 2021b).

The most likely explanation for this recent recovery of *Ae. pulcritarsis* in Austria is that this species has been overlooked in the past, since *Ae. pulcritarsis* uses tree-holes as breeding habitats, and these sites are not well investigated in most parts of Austria. The climatic and habitat situation in Central Europe seems to be generally suitable for *Ae. pulcritarsis*, because this species was described in the recent past for all neighbouring countries of Austria except Switzerland (Kampen et al. 2017; Robert et al. 2019). Since *Ae. pulcritarsis* has not been found earlier despite the longstanding mosquito monitoring programme in this area (mosquito monitoring A), this species is probably very rare in Lower Austria.

Although its main distribution is in the Mediterranean area, *Ae. pulcritarsis* can be found in many countries in Central Europe. The northern border of the distribution area of this species is marked by countries located in the same latitude or even further north than Austria, e.g. France, the Czech Republic or Slovakia (Robert et al. 2019). However, in those northern fringe areas, *Ae. pulcritarsis* is expected to occur at much lower abundances than in its main distribution area. To our knowledge, there are no reports showing or indicating that *Ae. pulcritarsis* has, due to recent environmental or climatic changes, dispersed farther north from its known distribution area. However, due to the lack of detailed information on this species’ distribution in Europe, the possibility of active spread of *Ae. pulcritarsis* from neighbouring countries because of changing climatic and environmental conditions cannot be ruled out. An introduction via human-made containers (such as tyres) also seems very unlikely, as *Ae. pulcritarsis* does not usually use these as breeding habitats. To our knowledge, *Ae. pulcritarsis* has never been associated with dispersal via road or air transport. Although one specimen was found at the Vienna airport, the occurrence at the second location makes an introduction via air travel unlikely. However, more detailed studies on the distribution of *Ae. pulcritarsis* are needed to confirm our assumption that this species is indeed native to Austria.

*Aedes pulcritarsis* is anthropophilic and presumably also zoophilic, and it is known to feed during daytime (Nikookar et al. 2010; Becker et al. 2020). However, this species seems to be rare in Austria, and its distribution is likely limited by its specialized breeding sites in woodlands with mature trees, located in forested mountainous areas with high annual precipitation (Müller et al. 2012). This reduces contact with people and thus its role as a nuisance species to humans. Currently, there is no information available on its vector status.

In combination with data from other European countries, the results presented here will contribute to the investigation of the northern distribution limit of *Ae. pulcritarsis* and possible changes to it. These first findings of a new mosquito species for Austria also suggest that the culicid fauna in Austria is still insufficiently documented and that further mosquito species, especially such with specialized and little studied breeding sites such as tree-holes, remain to be discovered.
